# Effect of Interfacial Lubrication between Rubber and Metal on Reducing Mixer Chamber Wall Wear in Mixing Process

**DOI:** 10.3390/polym14173473

**Published:** 2022-08-25

**Authors:** Yiren Pan, Zhihua Sui, Yihui Chen, Yi Pan, Shaoshu Tang, Chuansheng Wang, Wenwen Han

**Affiliations:** 1 College of Electromechanical Engineering, Qingdao University of Science and Technology, Qingdao 266061, China; 2 National Engineering Laboratory of Advanced Tire Equipment and Key Materials, Qingdao University of Science & Technology, Qingdao 266061, China

**Keywords:** interfacial lubrication, antifriction agent, effective friction, wear

## Abstract

This paper focused on adding a suitable lubrication effect at the interface between the rubber and mixer chamber wall on reducing the surface wear rate of the mixer chamber wall. In the research process, the contact model between the compound and internal mixer chamber wall was simplified to the pin-on-disc experimental model. The experimental results showed that the friction coefficient and the metal surface wear rate of the mixer chamber were reduced (by approximately 24%) by adding an appropriate amount of antifriction agent in the mixing process, while the comprehensive properties of the compound showed an improvement trend. By analyzing the surface elements of the rubber compound, the MoS_2_ with an anti-wear effect on the surface of the rubber compound can form a lubrication mechanism between the rubber, filler, and mixer chamber wall metal. Combined with the result of the comprehensive properties of rubber, which showed that although the appropriate amount of antifriction agent formed a lubrication protection mechanism between the rubber and the inner mixing chamber wall, the mechanism did not affect the friction behavior required for mixing. The study can effectively enhance the effective friction mixing and reduce the wear and power consumption of the mixing chamber caused by excess friction during the mixing process.

## 1. Introduction

Rubber products are widely used in the tire industry, however, due to the poor basic performance of the pure rubber matrix, it is often necessary to mix other fillers to improve its performance, especially the addition of a reinforcement system filler, which can significantly improve the performance of products [1]. Unlike carbon black, which relies on petroleum resources, silica is a green filler. Adding silica to tire products can reduce the rolling resistance and fuel consumption. With the gradual recognition of the advantages of silica, the content of silica in the tire formula and scope of application has increased gradually [2,3]. Due to the high hardness, silica is subjected to pressure from the upper top bolt and rotor during mixing, resulting in friction with the inner mixing chamber wall, which leads to the surface wear of the inner mixing chamber wall. In silica mixing with a coupling agent, a mixer chamber is used more as a reactor than as a mixer for the salinization reaction, and by-products of this reaction such as alcohols, water, and separated sulfur [4] can also enter damaged or worn areas and erode the softer underlying metal. Therefore, the products in the later stage of mixing will aggravate the wear caused in the early stage of mixing [5,6].

With the rapid development of modern technology, the energy consumed by friction contact accounts for about 30% of the total energy consumption in the world. Similarly, in the mixing process, with the addition of rubber and filler, the filling amount in the mixer chamber increases, and the rubber and filler create friction with the mixer chamber wall [7]. As the mixing continues, the increasing mixing temperature leads to the change in the rubber viscoelasticity, and the filler is continuously refined and dispersed by the tension, extrusion, and shear between the rotor and the mixer chamber, resulting in adhesion friction, lag friction, and filler friction. During the mixing process, the friction behavior causes wear on the mixing chamber wall. When the equipment runs for a long time, the surface wear of the metal is intensified, resulting in surface cracks [8,9]. To prevent the increase in the surface wear of the mixing chamber wall to prolong the service life of the mixing chamber, the wear resistance is usually improved by a heat treatment process or by adding a surface coating [10]. However, the heat treatment process and coating improvement are quite complex processes. After surface coating or a mixer surfacing overhaul, there will be poor bonding between the coating or surfacing layer and metal materials. In the process of increasing the hardness and wear resistance by heat treatment, cracks will expand due to alternating stress. There are extrusion changes and chemical reactions in the rubber mixing process that will also accelerate the expansion of surface cracks through physical and chemical effects.

In mechanical friction, to reduce the wear of the equipment surface caused by strong friction, lubrication is carried out between the contact surfaces [11,12,13]. There is residual industrial oil on the surface during the preparation of carbon black. During the mixing process, lubrication between the rubber and the internal mixing chamber wall can be generated, which can reduce the surface wear of the internal mixing chamber wall and protect the internal mixing chamber wall. However, silica is different. Although oil-extended styrene rubber will be added to the silica-based formula, the hardness of silica is very high, which will form dry friction with the internal mixing chamber wall during the mixing process and accelerate the surface wear of the internal mixing chamber wall [14]. Therefore, in order to prevent the surface wear of the mixer wall, an environmental protection oil will be added to the formula to form mixed lubrication between the rubber and the mixer chamber wall, which can reduce the wear of the mixing chamber wall during the mixing process and improve the mechanical performance [8,15]. Usually, an environmental protection oil is added when the mixing temperature reaches 110 °C, which is absorbed by rubber after mixing for a period, and the lubrication effect is reduced. It can be seen that this method cannot protect the internal mixing chamber wall in the early and late stages of silica mixing. At the same time, the excessive addition of environmental oil will also reduce the comprehensive mechanical properties of the rubber. Therefore, the friction characteristics of boundary lubrication in mechanical friction are introduced into the mixing process to reduce the metal surface wear caused by the friction between the polymer and metal [16,17].

In previous studies, it has been found that dialkyl dithiocarbamate (MoDTC) is one of the most widely used organic molybdenum friction modifiers. MoDTC has the best friction reducing effect at 250 °C, loses the anti-wear effect at 320 °C, and produces MoS_2_ with a higher content and better friction reducing and wear resistance at 125 °C. During the friction process, a physicochemical adsorption film with an antifriction and anti-wear effect can be formed on the friction surface, resulting in a better and lasting lubrication effect.

Therefore, this paper focused on the establishment of the interface lubrication contact (between compound and mixer chamber wall) in the mixing process before the silane coupling reaction, which was used to reduce the surface wear of the mixer chamber wall after the friction during the mixing process. According to the properties of the filler, the study of the interfacial lubrication was carried out by studying the chemical reaction of the antifriction agents to produce MoS_2_ with an antifriction and lubrication effect [18,19,20]. The changes in the soft friction and wear between the silica compound and mixer chamber wall were characterized by a CSM friction tester and three-dimensional morphology. Meanwhile, the effects of the addition of interfacial lubrication on the mixing effect and the comprehensive properties of the compound were studied. We also combined friction research with physical property research to explore the best amount of antifriction agents.

## 2. Materials and Methods

### 2.1. Materials

Thailand 20# Standard Rubber (TSR20) was the NR used in this study. Polymerized styrene-butadiene rubber (SBR; RC2557S) with a 57% vinyl content, cis-polybutadiene (BR9000), and sulfur (S) products were sourced from PetroChina Dushanzi Petrochemical Company, China. Carbon black (CB; N234) was obtained from Cabot, USA. Silica (Silica115MP) products were sourced from Rodia, while the silane coupling agent (Si69mix) was from Nanjing Shuguang Chemical Group, China. Meanwhile, 1,3-diphenylguanidine (DPG) and N-cyclohexyl benzothiazole-2-sulfenamide (CZ) were obtained from Guiechem, China. The protective and activation systems are qualified products approved by the industry.

### 2.2. Experiment Formula and Chemical Reaction Process

(1) Experiment formula

The formula used in this paper is shown in Table 1.

The sample was mixed using the Hackmie Machine developed by Qingdao University of Science and Technology. Three kinds of rubber were used in the formula to better disperse the silica filler and reduce the impact on the experiment. The fill factor and rotor speed was kept constant at 0.75 and 70 rpm, respectively. The discharge temperature was 155 °C. To ensure silica dispersion and achieve the temperature required for the silane coupling reaction, the reaction should be performed for 1.5 min at a mixing temperature of 145 °C–155 °C.

(2) Chemical reaction process

**Figure 1:**
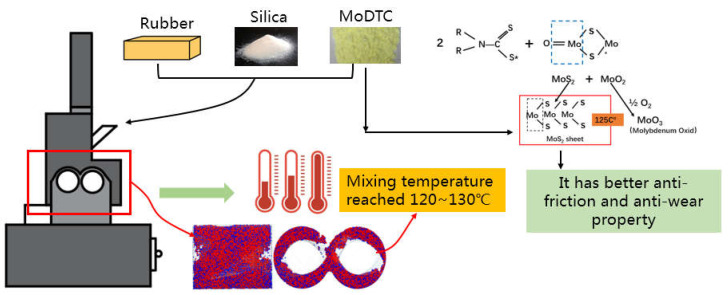
The chemical reaction process and interfacial lubrication products.

Figure 1 shows the chemical reaction after adding MoDTC in the whole mixing process. Since the corrosion wear of the mixer chamber wall caused by the products in the later stage of mixing (water and alcohol produced by silane coupling reaction) is based on the wear of silica compound on the mixer chamber wall in the early stage of mixing, the rubber test sample of the friction test sample was mainly obtained in the early and middle stage of mixing.

### 2.3. Experimental Process

(1) Comprehensive mechanical property test

➀ A rubber processing analyzer (RPA2000) was used to test the dynamic rheological properties of the compound. Strain scanning of the mixing rubber was performed under the following conditions: frequency of 1 Hz, the temperature of 60 °C, and strain range of 0.1; meanwhile, frequency scanning was performed at 60 °C, 7% strain, and 0.01 Hz frequency. ➁ Indentation hardness was determined using a durometer (Hardness Shore A). ➂ The dynamic strain was measured via the dynamic mechanical analysis (DMA). The tensile mode was used for temperature scanning, the temperature range was −65 °C–65 °C, the frequency was 10 Hz, and heating rate was 2 °C/min (GABOMETER−150, Germany). ➃ The tensile properties were determined using the Instron−3365 tensile force testing machine produced by Instron (Boston, America). ➄ Morphology analysis (SEM): Break the vulcanizate with liquid nitrogen, adhere it to the conductive tape, then fix it on the sample table and spray gold, and observe it under the scanning electron microscope (scanning electron microscope, JSM-7500F, Japan Electronics Corporation, Tokyo, Japan).

(2) Sample preparation

In the experimental process, the sample preparation was divided into two parts.

➀ Friction test sample: The chemical reaction of MoDTC produced MoS_2_ with an anti-wear effect at 125 °C, and formed the interfacial lubrication effect. Therefore, the samples were taken at the initial stage and the stage after MoS_2_ formation (the samples were obtained after a minute of mixing at 125–130 °C, which was helpful for the full decomposition reaction) during the mixing process, respectively. The extrusion device was used to ensure the relatively flat test surface of the rubber, which was used for the friction coefficient test between the rubber and metal;

➁ Rubber comprehensive performance test samples: Rubber comprehensive performance test samples need to be prepared according to the mixing process of the silica-based formula. Put the rubber into the mixer for 30 s, add small materials and 1/3 silica after 40 s, add 1/3 silica after 30 s, add residual silica after 30 s, and maintain for 1.5 min in the mixing temperature of 145–155 °C to promote the silane coupling reaction is fully carried out, and discharge at 155 °C.

(3) Friction and three-dimensional morphology test

**Figure 2:**
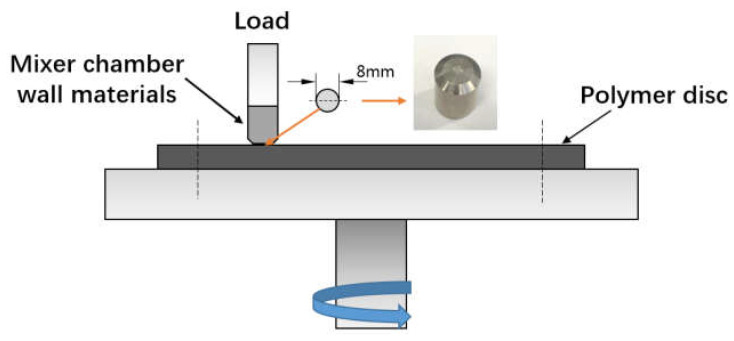
The friction test mode.

According to the contact mode between the rubber and mixer chamber wall in the mixing process, based on the principle of force interaction in Newton’s third law and area equivalence principle, we simplified the contact model between the rubber and metal into a pin-on-disc contact rotation experimental model, and the schematic contact mode of the frictional couple is shown in Figure 2. The upper block was the mixer chamber metal, the lower disc was the rubber compound. To ensure the accuracy of the data, the friction test was performed under the same rotation test conditions, which were calculated by the mixing process. The conditions of the mixing friction were mainly matched with the mixing process. In general, the mixing time of the silica rubber was 6 min, the speed was generally set to 40–60 rad/min. The pressure was calculated according to the equivalent conversion principle of the contact area of the mixing process and the area between the test contact surfaces, about 8 N. The test temperature was set according to the combined effect of the silica dispersion and the MoDTC chemical conversion temperature.

Before each test, the surface metal was wiped with alcohol, and the test points were marked on the metal surface to record the coordinates of the test points, which was convenient for the observation of the surface morphology after the friction test. The friction coefficient was continuously recorded by an online data acquisition system attached to the Tribometer of Anton Paar (short for CSM). The friction coefficient was the average value during each part of the friction test.

The surface morphologies of the metal and rubber samples before and after friction were observed using the three-dimensional topography instrument (Olympus 4500) to track the sliding trajectory and magnified the test point at the same position with a 20× objective lens. The measured area was about 640 µm × 640 µm, and the surface change of the wear rate, *Sa*, *Sp*, and *Sv* of the metal were calculated, and the calculations are shown in Equations (1)–(4) [21].

(1) $$Sa=\frac{1}{A}\int \int_{0}^{A}\left|Z(x,y)dsdy\right|$$

(2) Sp=maxz(x,y)

(3) $$Sv=\left|\max z(x,y)\right|$$

Wear rate = *V*/*Fnl*(cm^3^/Nm) (4)

where *V* is the wear volume loss (mm^3^); *L* is the sliding distance (mm) of very circle; *F* is the load (N); and *n* is the number of circles.

## 3. Results and Discussion

### 3.1. Average Friction Coefficient and Friction Coefficient Change

**Figure 3:**
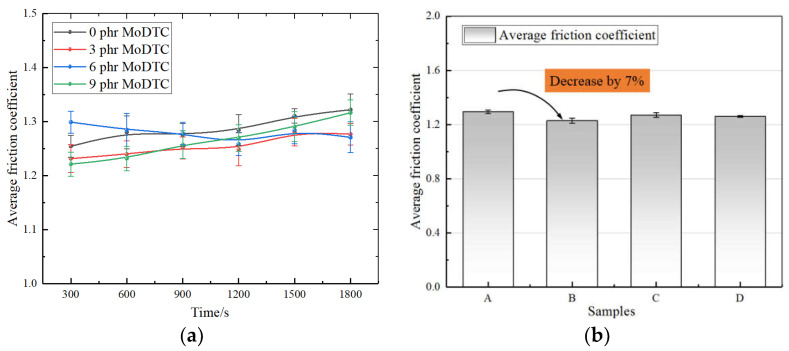
The comparison of the average friction coefficient and friction coefficient change between the four samples and metal. (**a**) Friction coefficient change. (**b**) Comparison of the average friction coefficient (A: 0 phr MoDTC; B: 3 phr MoDTC; C: 6 phr MoDTC;D: 9 phr MoDTC).

Figure 3 shows the average friction coefficient between the four samples and mixer chamber wall materials over the entire test, which showed that adding MoDTC could reduce the friction coefficient of both. The experimental setup was found to provide repeatable results, allowing for a clear comparison that showed the effects of MoS_2_ formed by different contents of MoDTC at 125 °C on the friction coefficient during silica compound mixing.

By comparing the friction coefficient between four samples and the mixer chamber wall during the mixing process, the friction coefficient gradually decreased with an increase in the MoDTC content. Compared to that of A without MoDTC, the average friction coefficient of B with 3 phr MoDTC decreased by 7%, while those of C–D decreased slightly. At the same time, the average friction coefficient of C and D showed an upward trend relative to B. It can be explained that MoDTC decomposed in the mixing process to produce MoS_2_ with lubrication, which produced the lubrication friction effect between the rubber and metal. In the mixing process, a proper average friction coefficient was an important mixing factor to improve the mixing efficiency and filler dispersion.

### 3.2. Wear Rate of Mixer Chamber Wall Materials

**Figure 4:**
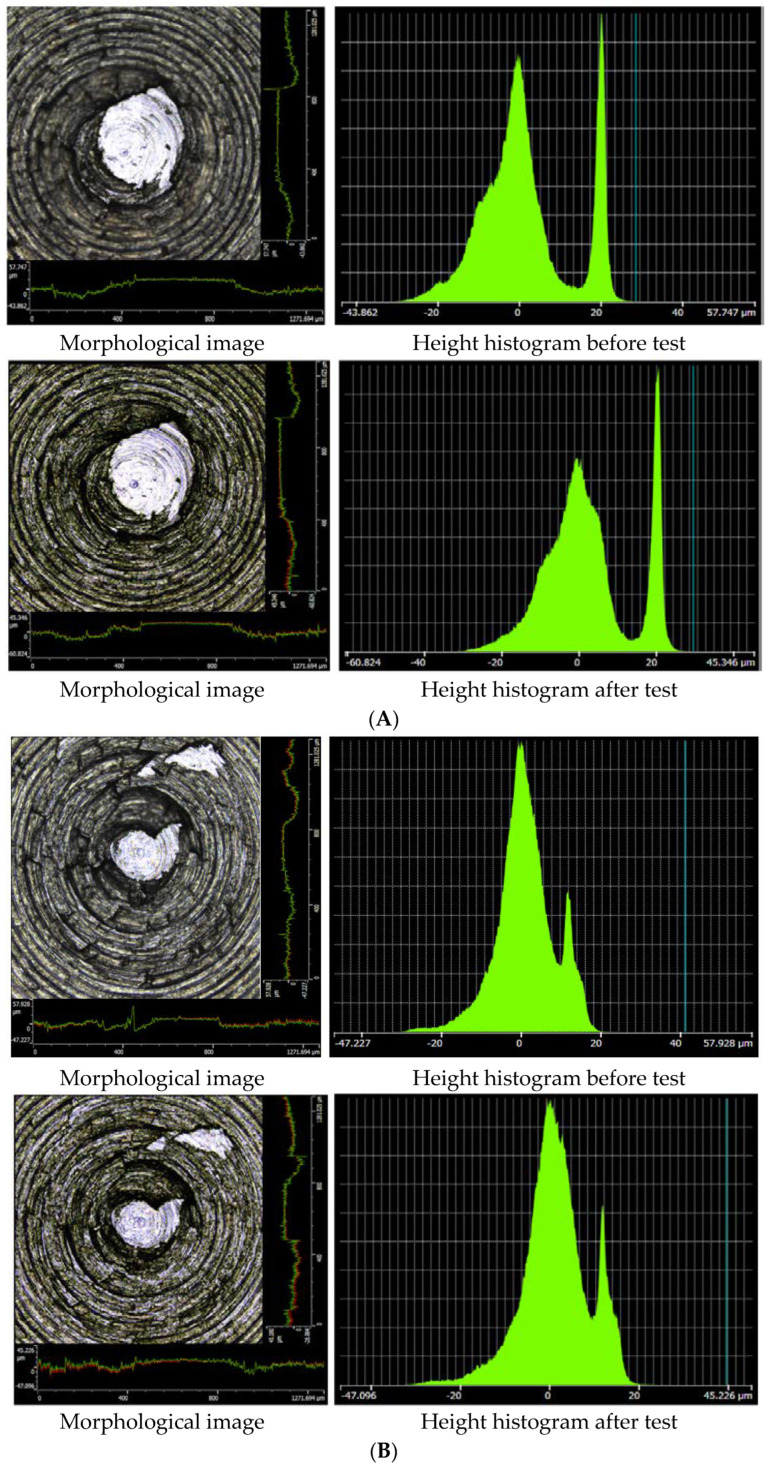

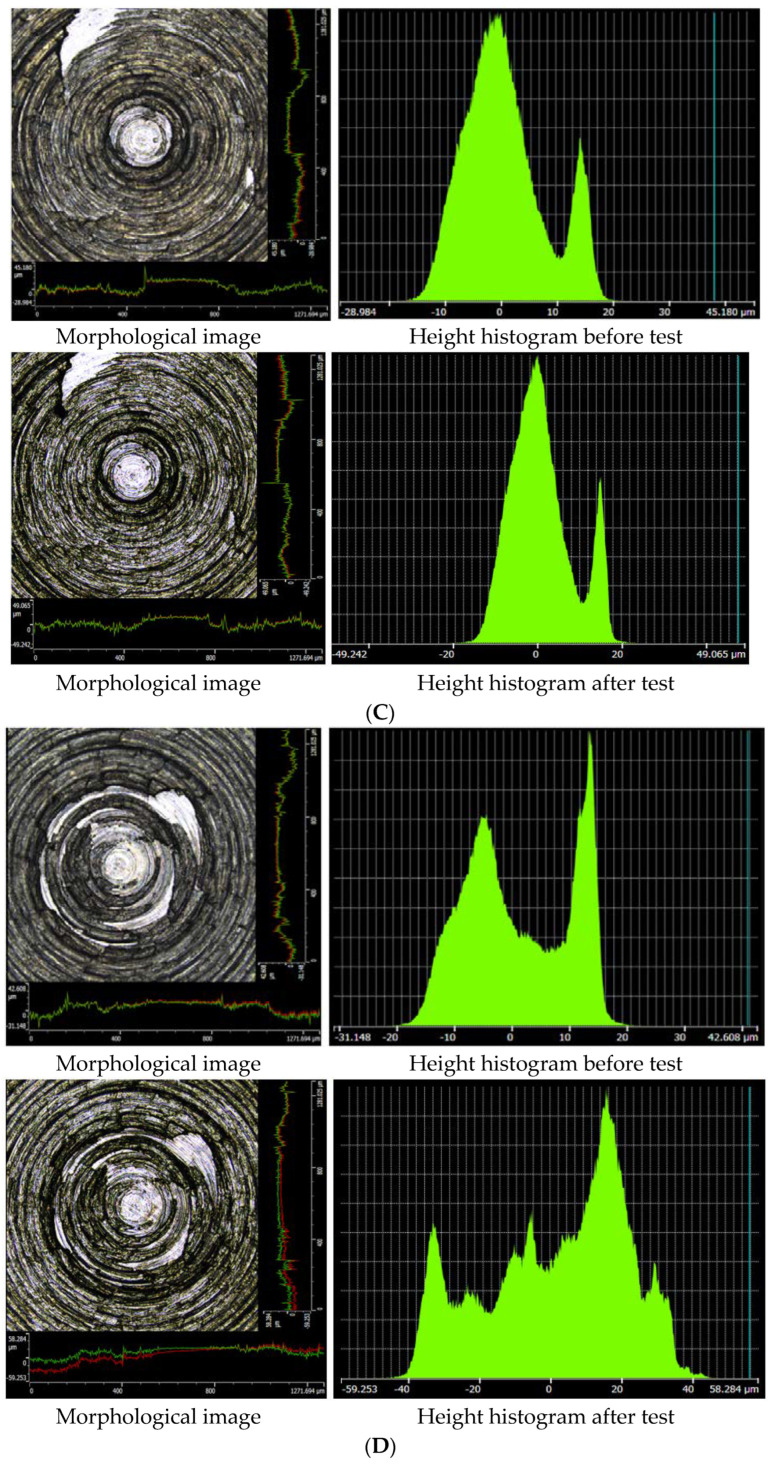
A comparison of the surface morphology changes in the mixer chamber wall (left is the morphological image, right is the height histogram ((**A**): 0 phr MoDTC; (**B**): 3 phr MoDTC; (**C**): 6 phr MoDTC; (**D**): 9 phr MoDTC).

Figure 4 shows the comparison of the mixer surface morphology and height histogram changes after the friction test between four samples under the test temperature of 125–135 °C. The change in the surface morphology of the mixer chamber wall before and after the friction test between sample B and metal was the smallest, and the change in the metal surface height also showed the same trend. However, the other three groups of experiments exhibited an evident wear phenomenon, and the height range of the metal surfaces changed significantly, which was especially true for samples C and D, the surface height histogram of the mixer chamber wall changed greatly.

**Figure 5:**
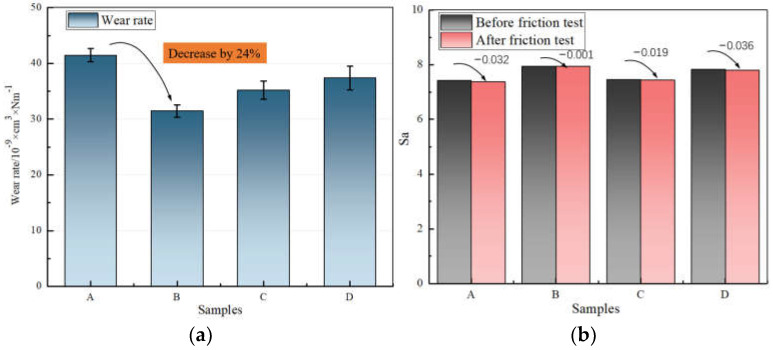
A comparison of the wear rate and *Sa* of the metal surface. (**a**) Wear rate and (**b**) comparison of *Sa* (A: 0 phr MoDTC; B: 3 phr MoDTC; C: 6 phr MoDTC; D: 9 phr MoDTC).

Figure 5 shows the comparison of the wear rate and *Sa* of the metal surface. Through the change in the metal surface wear rate, it was found tFat the wear rate of the mixer chamber wall decreased significantly after rubbing with the silica compound with 3 phr MoDTC, and the metal surface roughness remained unchanged. The friction between the other three samples and metal tended to produce a certain amount of wear and surface roughness; this was especially true for D with 9 phr MoDTC, in which the metal surface wear rate and surface roughness showed an upward trend. By comparing the friction coefficient and wear rate of the metal, it could be found that there was no linear relationship between the wear rate and friction coefficient (rubber and metal), that is, the change in wear rate was not necessarily the same as that of the friction coefficient. The phenomenon could be obtained from the friction behavior of samples C and D with the metal. After observing the friction between sample B and the metal, the change in the metal surface and friction coefficient showed the same downward trend. Through this phenomenon, we believe that the appropriate antifriction agent can reduce the wear rate on the metal surface while establishing the interface lubrication, thus achieving the desired effect.

It is well-known that the friction behavior in the mixing process will affect the dispersion of the filler, compound viscoelasticity, and chemical reaction of the rubber. Although it was found that sample B with 3 phr MoDTC showed a promising downward trend in the friction coefficient and wear rate after friction, the impact of this result on the properties of the compound was also a problem that we must pay attention to. The research results must ensure that they will not affect the comprehensive properties of the compound. The feasibility of reducing the surface wear rate of the mixer chamber wall through the filler chemical reaction can be determined by combining the comprehensive mechanical properties with the friction experimental results.

### 3.3. Comprehensive Mechanical Properties of the Compound with MoDTC

**Figure 6:**
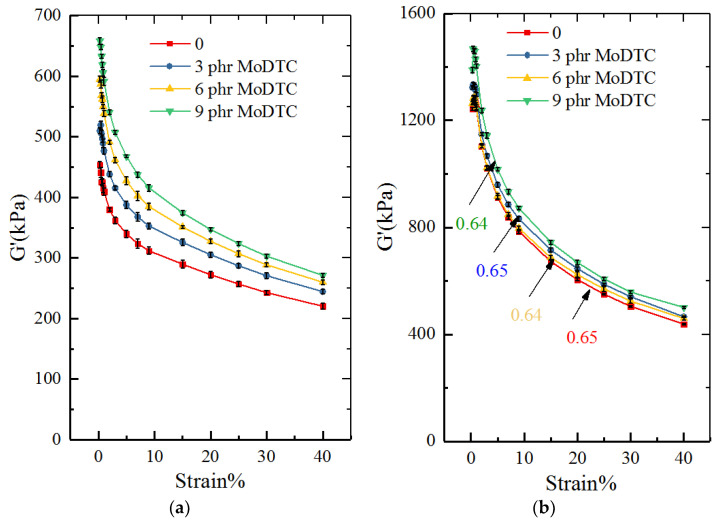
The Payne effect and silane coupling reaction degree of the RPA test. (**a**) Payne effect and (**b**) silane coupling reaction.

Figure 6 shows the Payne effect and silane coupling reaction degree of the RPA test. Figure 6a depicts the effect of MoDTC on the silica dispersion and the network of silica/rubber compounds. It is known that the dynamic strain depends on the energy storage modulus of the rubber filling, which exhibits a nonlinear response with the change in strain (called the Payne effect). The Payne effect in RPA is usually used to reflect the filler dispersion; the greater the Payne effect, the larger the filler network. Furthermore, the larger the G ‘Max/G’ min ratio, the better the silica dispersion. By comparing the Payne curves and the calculated G’max/G’min ratios of the four samples in Figure 6a, it was found that with an increase in the amount of MoDTC, the Payne effect curve and G’max/G’min ratio showed an upward trend, thereby indicating that the silica dispersion was improved.

The silane reaction between the silica and silane coupling agent can improve the dispersion of silica and its compatibility with rubber. Therefore, the modified silica amount governs the rubber properties. The degree of the silane coupling reaction is an important parameter to measure the amount of silica modified. In order to test the silane coupling degree of silica in the mixing process, it was characterized by the Payne effect. During the Payne test, the intermolecular Brownian motion can be promoted by further heating due to the breakage of filler–filler interactions. Meanwhile, silica without the silane coupling agent is more likely to agglomerate due to its surface hydroxyl groups. Therefore, the coupling degree of silica can be calculated by the deformation curves obtained by adding (or not adding) the silane coupling agent (Figure 6b)). According to the curve calculation, adding MoDTC can improve the silane coupling reaction and silica dispersion, and the ratio of the silane coupling reaction agents was enhanced by 5%. In light of this phenomenon, when the mixing temperature reaches 125 °C, MoDTC would produce MoS_2_, which would lead to lubrication and generate a film to push the rubber to flow and promote the dispersion of silica.

**Figure 7:**
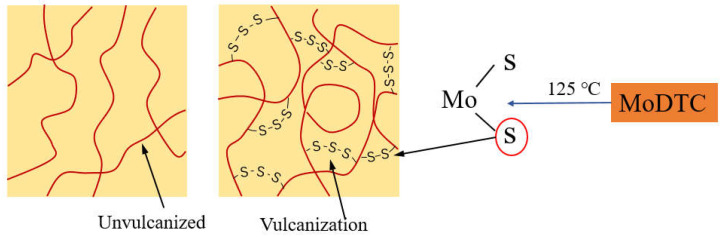
The process of MoS_2_ participated in the vulcanization.

The vulcanization data of the four samples are shown in Table 2. ML can be used to represent the fluidity of rubber materials. The higher the ML value, the lower the fluidity. MH is the max torque, which can be used to characterize the shear modulus, hardness, and tensile strength of the compound. MH-ML can characterize the cross-linking density of rubber. According to the vulcanization data of four samples, with an increase in the MoDTC amount, the fluidity of the compound decreased. Combined with the Payne effect data, the low fluidity of rubber was mainly caused by the poor dispersion of the filler. The value of MH-ML showed an upward trend and then a downward one, which indicated that the crosslinking density increased with appropriate MoS_2_ content. Comparing A without MoDTC and B with 3 phr of MoDTC, the cure time decreased with an increase in curing speed. The main reason for this phenomenon was that the sulfur in MoS_2_ also participated in the formation of the vulcanization grid during vulcanization (the process is shown in Figure 7). However, excessive MoS_2_ would accumulate and the sulfur was unable to form a vulcanization grid with the silica and rubber, preventing silica from being linked with the rubber molecules, resulting in reduced crosslinking density. The experimental results also showed that the vulcanization period decreased after adding MoS_2_, which was conducive to improving the vulcanization efficiency.

The hardness of the samples exhibited minor changes after the addition of MoDTC, which indirectly indicates that the interface lubrication effect produced by adding the appropriate MoDTC does not affect the friction behavior needed to improve the dispersion of silica in the mixing process. Furthermore, the 10% tensile modulus had a strong relationship with hardness. The 100% tensile modulus could be used to characterize the rubber–filler interactions; B exhibited the largest 100% tensile modulus, which showed that the addition of 3 phr of MoDTC could promote the silica dispersion in rubber and link it with the rubber molecular chain. The 300% tensile modulus could represent the composite action in both the filler–rubber and rubber–rubber interactions; B with the largest 300% tensile modulus showed a good silica dispersion and a high-curing crosslinking density. Since MoDTC decomposed MoS_2_ at 125 °C, the sulfur in MoS_2_ participated in the vulcanization process (the process is shown in Figure 7) and formed a more cross-linked network structure in the vulcanization process. Therefore, the density of the vulcanization network structure increased and the 300% tensile modulus showed an upward trend. The test results indicated that the compatibility between the silica and rubber and the crosslinking density silica compound could be increased by adding the appropriate MoDTC in the silica system.

**Figure 8:**
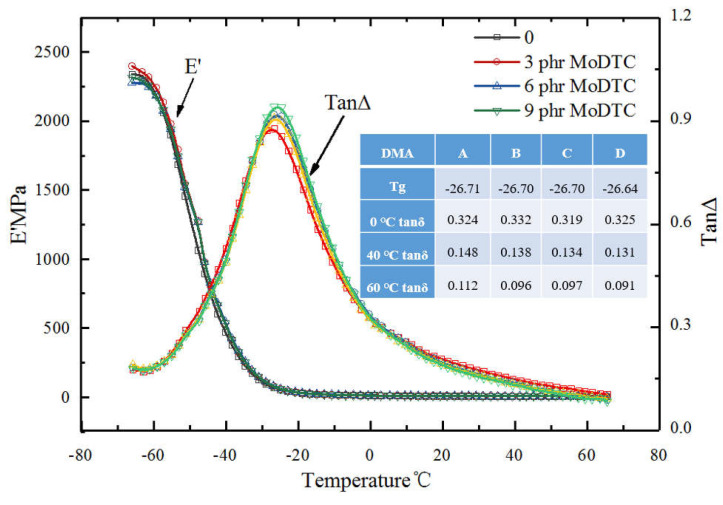
The E’ and TanΔ of the DMA test.

Figure 8 shows the E’ and TanΔ of the DMA results of the four samples. There was no problem of poor compatibility in the DMA curve by adding MoS_2_ (double-peak phenomena). To carefully study the change in the dynamic mechanical properties of MoS_2_ generated in the mixing process, a comparative analysis was conducted on 0 °C @ tanδ, 40 °C @ tanδ, and 60 °C @ tanδ. Generally, 0 °C @ tan δ and 40 °C @ tan δ area can represent the wet slip resistance of rubber, while 60 °C @ tan δ can relate to the rolling resistance performance of rubber. Furthermore, the MoDTC addition had a negligible effect on the glass transition temperature of the compound. The performance of B with 3 phr of MoDTC was better than that of the other three samples. The tanδ @ 0 °C and tanδ @ 40 °C of B were relatively larger than that of the other samples, while tanδ @ 60 °C was relatively lower than that of A with no MoDTC. The test results revealed that adding an optimum amount of MoDTC could reduce the rolling resistance of the tire and improve the wet sliding resistance effect. We believed that although the released MoS_2_ was mainly used to reduce the wear, it improved the fluidity of the rubber and the dispersion effect of the filler in the rubber to a certain extent; furthermore, the dispersion effect of silica directly affected the dynamic mechanical properties of the rubber.

### 3.4. Interfacial Lubrication Mechanism of MoS_2_ during Silica Compound Mixing

**Figure 9:**
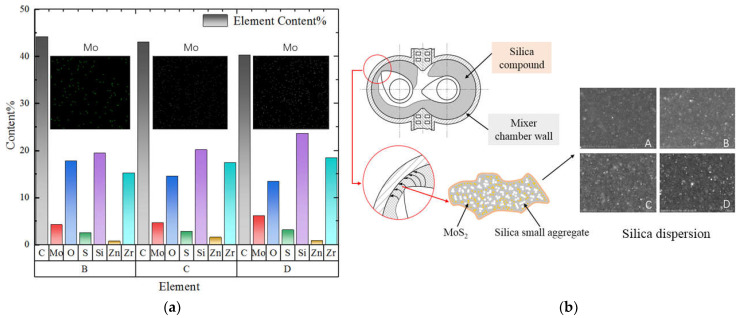
The element dispersion content and interfacial lubrication mechanism of MoDTC. (**a**) Element dispersion content. (**b**) Interfacial lubrication mechanism of MoDTC (A: 0 phr MoDTC; B: 3 phr MoDTC; C: 6 phr MoDTC; D: 9 phr MoDTC).

Figure 9 depicts the results of the elemental analysis of SEM, and the elemental contents and distributions. The main purpose of the test was to verify the influence of MoS_2_ produced by MoDTC at 125 °C during mixing on the silica dispersion, determine the distribution of Mo, and elucidate the increase in the sulfur content for every 3 phr of MoDTC added. The dispersion images revealed that more Si elements were aggregated with an increase in the MoDTC content, and that accumulation of the elements could produce a large agglomeration of particles. Furthermore, it can be explained that the MoDTC extracts lubricated MoS_2_ when the mixing temperature reached 125 °C and that the silane coupling reaction had already begun at this stage while the crushing and dispersion of silica aggregates were still in progress. However, in the mixing process, friction was a crucial factor to promote the mutual infiltration of the rubber and filler and improve the mixing efficiency. Based on the distribution of the Si and SEM images, it was found that excessive MoS_2_ would reduce the advantage of friction in the mixing process and affect the final filler dispersion effect. The Mo elements were dispersed relatively evenly in B, while the Mo elements in C and D were accumulated. The experimental results showed that adding too much MoDTC in the mixing process resulted in the accumulation of MoS_2_.

This phenomenon showed that the excessive addition of MoDTC will weaken the dispersion of silica in the mixing process and also affect the dispersion of MoS_2_. The main reason was that the excessive interfacial lubrication of MoS_2_ will weaken the tensile and shear effects conducive to the dispersion of silica. However, due to the hardness and easy agglomeration of silica, when it passed through the gap between the rotor and the internal mixing chamber wall, it was easier to destroy the established interface lubrication state, thereby accelerating the wear of the mixing chamber wall. Therefore, the element distribution further showed the importance of an appropriate number of copies of MoDTC to establish a good interface lubrication effect.

## 4. Conclusions

This paper focused on the addition of an antifriction agent in the mixing process to establish the interfacial lubrication effect between the rubber and internal mixing chamber wall, which was used to improve the effective friction behavior in the mixing process and reduce the surface wear rate of internal mixing chamber wall. At the same time, the concept of effective friction mixing was also proposed. According to the principle of area equivalence, the contact model between the rubber compound and internal mixing chamber wall in the mixing process was simplified into the pin-on-disc contact rotation model, which is convenient for tracking and observation. The friction test, wear rate analysis, and the comprehensive mechanical property data of the silica compound showed that after adding 3 phr of MoDTC decomposed into MoS_2_ in the mixing process, the friction coefficient between the rubber and metal decreased by 7%, and the wear rate of the metal surface decreased by 24.13%. Compared with the other three samples, the wear reduction effect was more obvious. It can be explained that adding MoS_2_ generated by MoDTC in the mixing process will produce an interfacial lubrication effect between the rubber and the internal mixing chamber wall. The analysis of the comprehensive mechanical properties of the compound showed that the dispersion effect of silica, the comprehensive mechanical properties of rubber, the vulcanization crosslinking density, and the dynamic mechanical properties obtained by using the DMA test showed a slight increase with the addition of 3 phr MoDTC. The comprehensive analysis found that the addition of the appropriate MoDTC in the mixing process reduced the friction coefficient and protected the internal mixing chamber wall, but had no effect on the comprehensive properties of the compound, indicating that appropriate antifriction agents can improve the effective friction mixing behavior. This work provides a new method for prolonging the service life of the internal mixing chamber from a chemical point of view, puts forward a new mixing concept, and provides a new research direction for improving the mixing process and efficiency.

## Figures and Tables

**Table 1:** The sample formula (unit: phr).

Raw Material	A	B	C	D
BR9000	25.5	25.5	25.5	25.5
RC2557S	81.81	81.81	81.81	81.81
TSR20	15	15	15	15
N234	10	10	10	10
Silica115MP	60	60	60	60
Si69mix	7.2	7.2	7.2	7.2
DPG	1	1	1	1
MoDTC	0	3	6	9
S	1.3	1.3	1.3	1.3
CZ	1.8	1.8	1.8	1.8
Others	Protection system: 3.5 phr; Activation system: 4 phr			

**Table 2:** The vulcanization data of the compounds.

Test List	A	B	C	D
ML(Nm)	1.82	2.02	2.21	2.35
MH(Nm)	17.53	18.03	17.05	16.92
MH-ML(Nm)	15.91	16.01	14.84	14.57
tc90(min)	16.51	9.96	9.55	9.17
Hardness (Shore A)	61	61.5	61	61.5
10% Tensile Modulus (MPa)	0.74	0.76	0.66	0.76
100% Tensile Modulus (MPa)	2.11	2.56	2.46	2.53
300% Tensile Modulus (MPa)	7.64	9.45	8.62	9.29
Tensile Strength (MPa)	15.37	15.96	13.85	13.64
Elongation at Break (%)	465.37	481.80	421.03	393.16

## Data Availability

Not Applicable.
